# Analysis and experimental validation of genes and their transcription factor prediction in contused rat spinal cord at the intermediate phase

**DOI:** 10.18632/aging.205912

**Published:** 2024-06-08

**Authors:** Zhongju Shi, Tuo Fang, Baoyou Fan, Jun Ma, Jianhao Wang, Shiqing Feng

**Affiliations:** 1 Department of Orthopaedics, Tianjin Medical University General Hospital, Tianjin, P.R. China; 2 International Science and Technology Cooperation Base of Spinal Cord Injury, Tianjin Key Laboratory of Spine and Spinal Cord, Tianjin, P.R. China

**Keywords:** spinal cord injury, intermediate phase, differentially expressed genes, transcription factors, TFAM

## Abstract

The intermediate phase of spinal cord injury (SCI) serves as an important target site for therapeutic mediation of SCI. However, there is a lack of insight into the mechanism of the intermediate phase of SCI. The present study aimed to investigate the molecular mechanism and the feasible treatment targets in the intermediate phase of SCI. We downloaded GSE2599 from GEO and identified 416 significant differentially expressed genes (DEGs), including 206 downregulated and 210 upregulated DEGs. Further enrichment analysis of DEGs revealed that many important biological processes and signal pathways were triggered in the injured spinal cord. Furthermore, a protein-protein interaction (PPI) network was constructed and the top 10 high-degree hub nodes were identified. Furthermore, 27 predicted transcription factors (TFs) and 136 predicted motifs were identified. We then selected insulin-like growth factor 1 (IGF1) and its predicted transcription factor, transcription factor A, mitochondrial (TFAM) for further investigation. We speculated and preliminarily confirmed that TFAM may regulate gene transcription of IGF1 and effected alterations in the function recovery of rats after SCI. These findings together provide novel information that may improve our understanding of the pathophysiological processes during the intermediate phase of SCI.

## INTRODUCTION

The occurrence of spinal cord injury (SCI) is a profoundly detrimental event that can seriously affect human life, and result in abnormal or absent motor and sensory function [1–3]. The annual incidence rate of SCI ranges from approximately 13.3 to 45.9 cases per million individuals, and the number is increasing every year [4, 5]. Therefore, SCI has led to significant burden on human society, with high rate of mortality and dependency, and treatment of SCI has been a major concern in the medical field [6, 7]. However, spontaneous recovery is limited, and there is still no generally accepted treatment and pharmacological therapies for the injured spinal cord [1, 6, 8].

Traumatic SCI results in pathophysiologic changes that range from minutes to years after the injury and include primary (the mechanical injury) and secondary injury (the delayed physiological cascade) [9]. During the intermediate phase (2 weeks to 6 months after SCI), the continued maturation of the astrocytic scar constitutes a pivotal process and leads to conduction deficits [10–12]. Even if a lot of attempts and endeavors were made to eliminate glial scar, the therapeutic benefits have been limited [13–15]. Furthermore, the intermediate phase is also characterized by cyst formation and regenerative axonal sprouting [10, 11]. Thus, the intermediate phase of SCI serves as an available and important target site for therapeutic mediation of SCI, and it’s essential to find the key therapeutic target for SCI. However, there is a lack of an insight into the mechanism of the intermediate phase of SCI, and it is necessary to identify the crucial genes and pathways during this phase of SCI.

A global analysis is essential to explore the molecular mechanisms and therapeutic strategies for SCI, and in the past years, microarray and RNA-sequencing analysis have provided important insights into gene expression changes after SCI [16–18]. Moreover, many recent studies did not capture the full extent of the intermediate phase of SCI, and the absence of key markers of SCI has hampered the therapy development, and finding appropriate target genes and pathways will allow researchers and clinicians to design innovative therapeutic strategies for the treatment of SCI. Therefore, potent therapeutic target genes and pathways of SCI need to be explored.

In this study, we first analyzed the expression of genes in spinal cord samples from GEO database (GSE2599) and caught sight of the top 10 up-regulated and down-regulated genes at 35 days following SCI. We conducted Gene Ontology (GO) and Kyoto Encyclopedia of Genes and Genomes (KEGG) enrichment pathway analysis to elucidate the molecular mechanisms underlying differentially expressed genes (DEGs)-induced pathophysiological changes. Furthermore, we determined the top 3 modules that may reveal pivotal functions in the intermediate phase of SCI, and a bioinformatic analysis was also performed on them. We also identified the top 5 key DEGs, and predicted their transcription factors (TFs). Then the primary research is carried out. These results could improve the understanding of the intermediate phase of SCI and optimize therapies for SCI.

## RESULTS

### Identification of the DEGs

**Figure 1:**
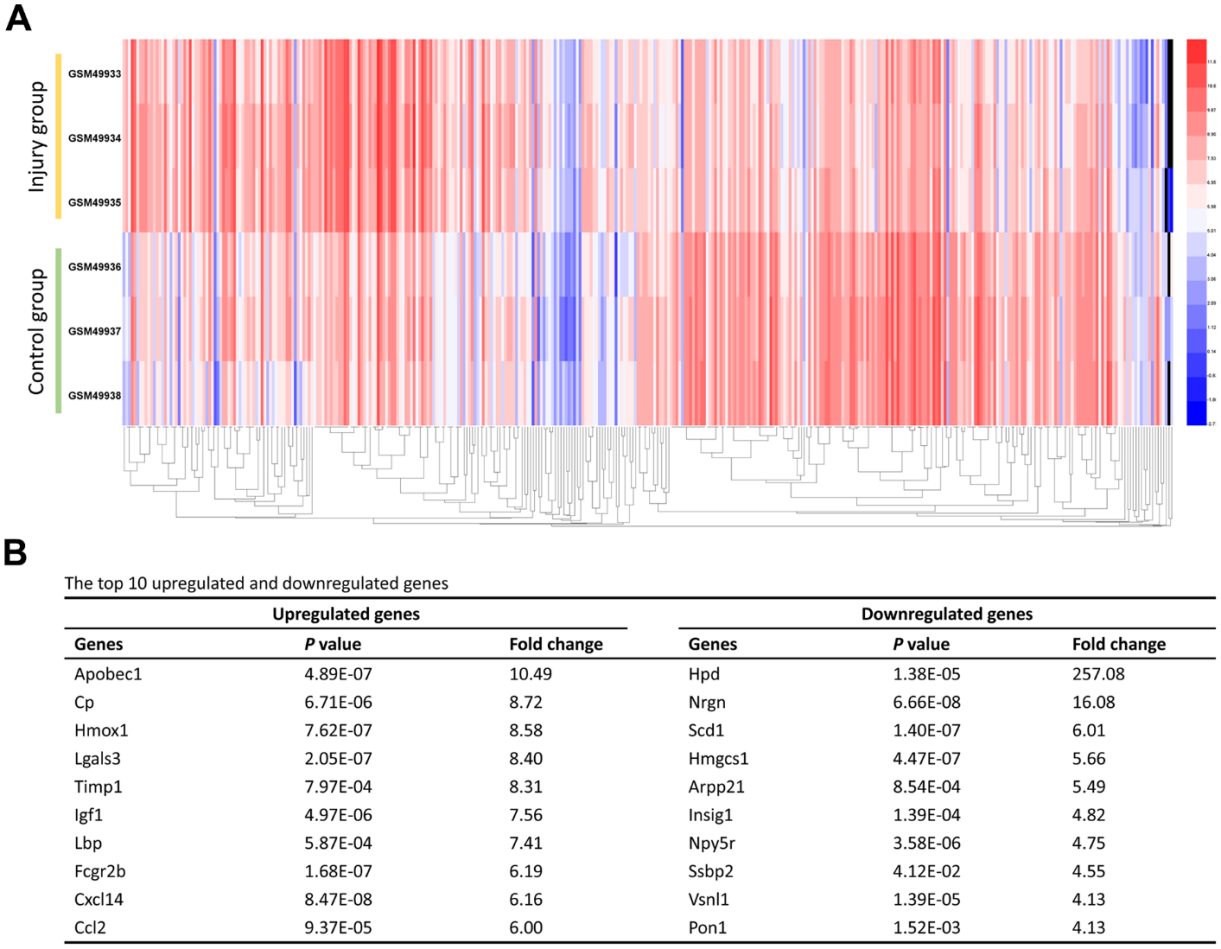
**Identification of the DEGs.** (**A**) The heat map of the 416 DEGs. Changes in genes expression (P < 0.05, logFC > 1.0 or logFC < -1.0) are illustrated by a heat map. Blue indicates a relatively low expression and red indicates a relatively high expression. (**B**) The top 10 upregulated and downregulated genes.

We initially retrieved gene expression profile (GSE2599) from the GEO database. A total of 416 DEGs expressed in contusion injured spinal cord samples were extracted from the database. Expressions of 206 genes (49.52%) were down-regulated and 210 genes (50.48%) were up-regulated in comparison to uninjured spinal cord samples. Subsequently, the heat map of the DEGs was generated to visualize their expression levels across various samples (Figure 1A). Furthermore, the top 10 up-regulated and down-regulated genes were list in Figure 1B.

### GO term and KEGG pathway enrichment analysis

**Figure 2:**
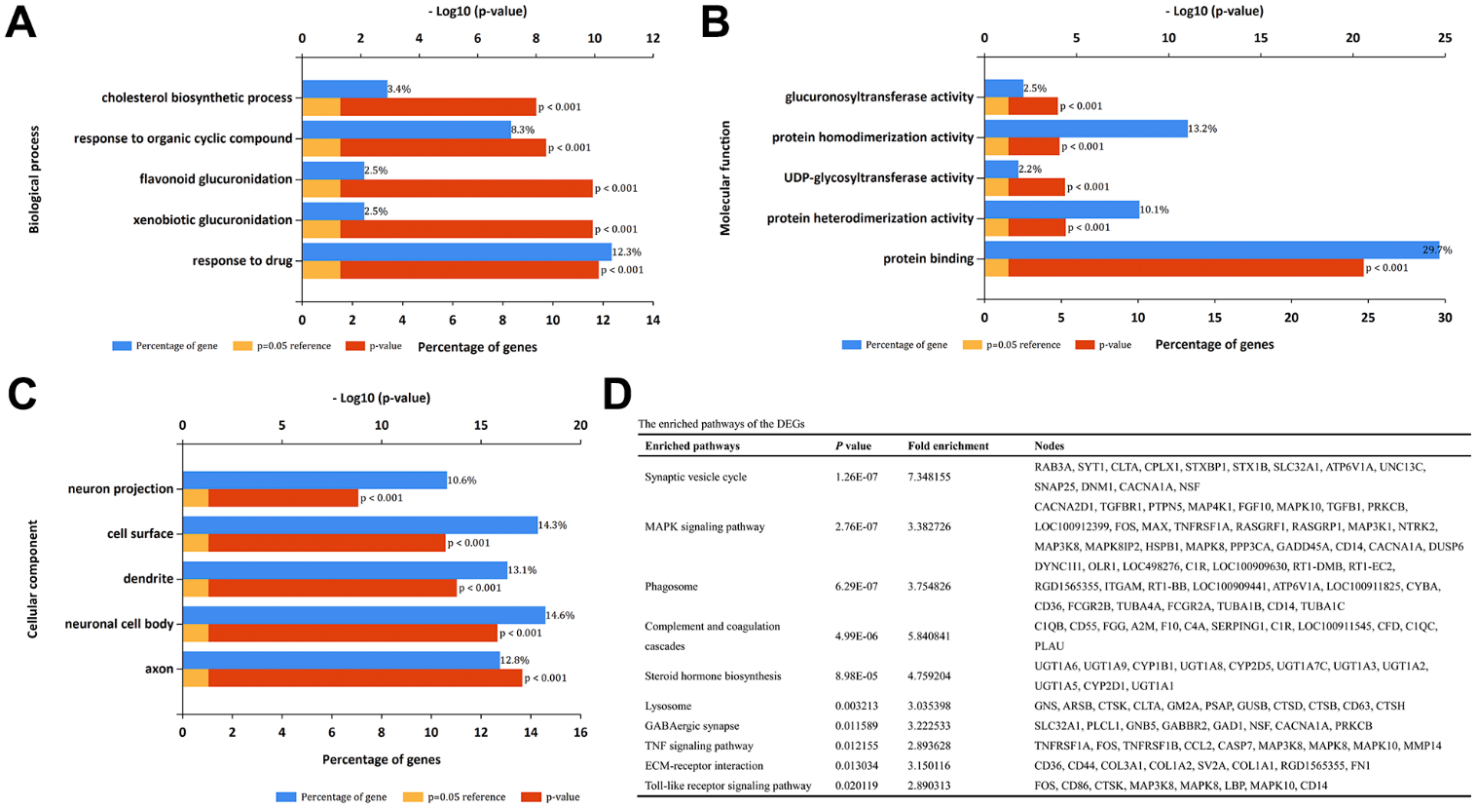
**GO term and KEGG pathway enrichment analysis.** (**A**) Top 5 enrichment terms of biological processes. (**B**) Top 5 enrichment terms of molecular function. (**C**) Top 5 enrichment terms of cellular component. (**D**) The enriched pathways of the DEGs.

DAVID was used to perform GO term and KEGG pathway enrichment analysis for the identified DEGs. GO analysis results showed that for biological processes (BP), the DEGs were significantly enriched in cholesterol biosynthetic process, response to organic cyclic compound, flavonoid glucuronidation, xenobiotic glucuronidation and response to drug (Figure 2A). For molecular function (MF), the DEGs exhibited significant enrichment in the processes related to glucuronsyltransferase activity, protein homodimerization activity, UDP-glycosytransferase activity, protein heterodimerization activity and protein binding (Figure 2B). For cell components (CC), the DEGs exhibited significant enrichment in the processes related to neuron projection, cell surface, dendrite, neuronal cell body and axon (Figure 2C). Moreover, the top ten KEGG pathways were listed in Figure 2D, including synaptic vesicle cycle, MAPK signaling pathway, phagosome, complement and coagulation cascades and so on.

### PPI network of the DEGs and core genes in the PPI network

**Figure 3:**
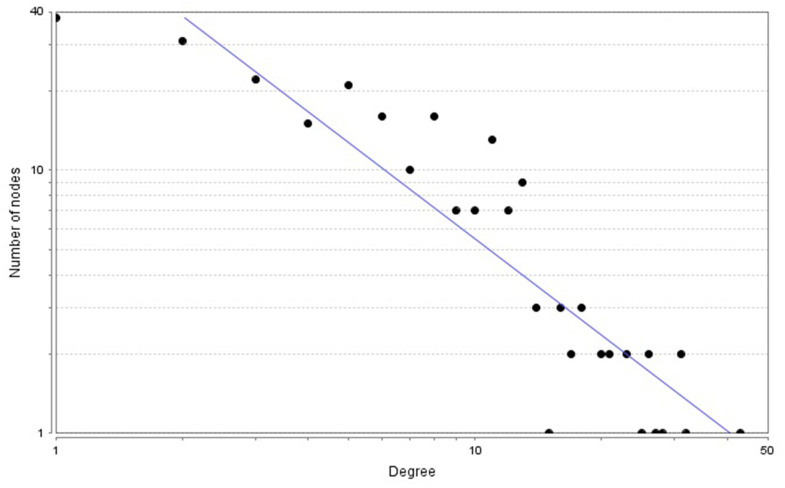
Power-law distributions of node degrees for the PPI network.

**Figure 4:**
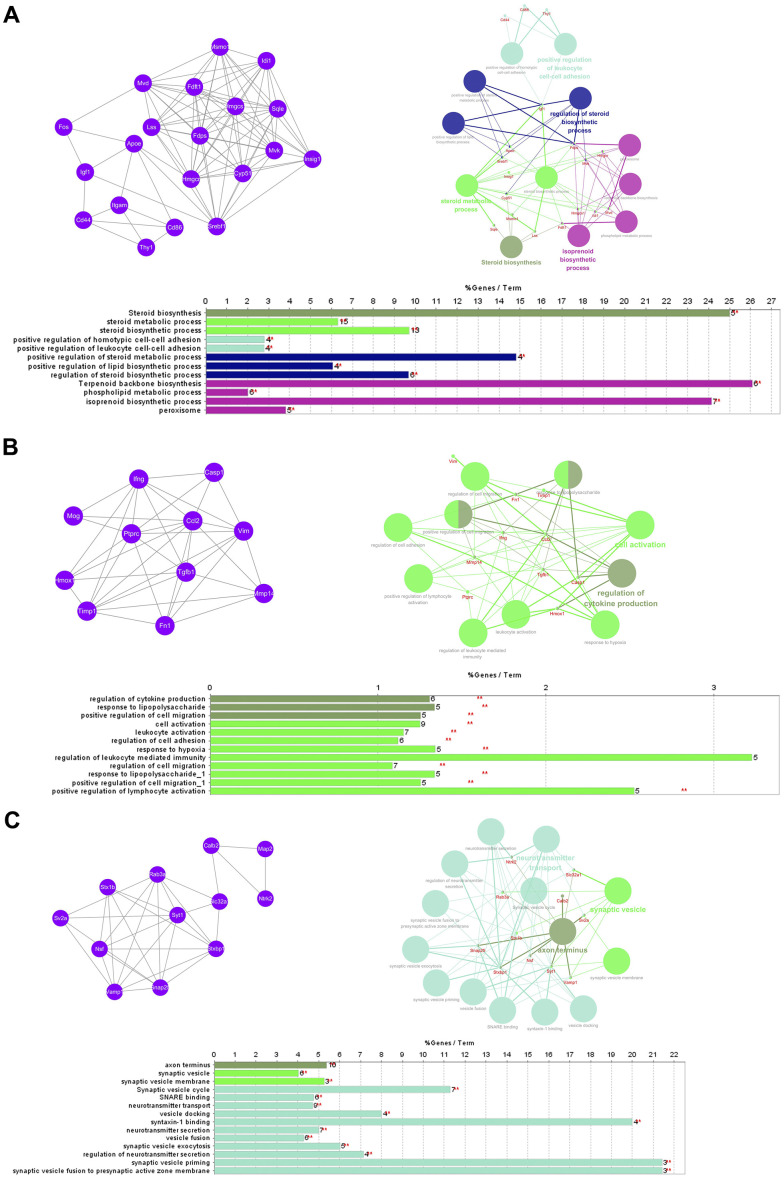
**GO term and KEGG pathway enrichment analysis of the top 3 modules.** (A) Module 1. (B) Module 2. (C) Module 3.

The PPI network comprised 239 nodes and 846 edges, as inferred from the information available in the STRING database. Here, nodes represent DEGs, while edges signify the interactions between these DEGs. The top 10 high-degree hub nodes consisted of FBJ osteosarcoma oncogene (FOS), TIMP metallopeptidase inhibitor 1 (TIMP1), C-C motif chemokine ligand 2 (CCL2), vimentin (VIM), insulin-like growth factor 1 (IGF1), calbindin 2 (CALB2), apolipoprotein E (APOE), protein tyrosine phosphatase, receptor type, C (PTPRC), interferon gamma (IFNG) and transforming growth factor, beta 1 (TGFB1). Among these genes, FOS exhibited the highest node degree of 43. The core genes and their corresponding degrees are presented in Table 1. The node degrees for the total DEGs generally followed power-law distributions, as illustrated in Figure 3. Subsequently, the modules of the PPI network were screened using Cytoscape, and subsequent GO and KEGG enrichment analyses were conducted for the genes involved in the top 3 significant modules (Figure 4A–4C). The findings indicate that the DEGs in the top 3 modules were principally related to steroid biosynthesis, regulation of homotypic cell-cell adhesion, regulation of cytokine production, regulation of cell migration, leukocyte activation, lymphocyte activation, axon terminus, synaptic vesicle and neurotransmitter secretion.

**Table 1:** The core genes and their corresponding degree.

**Gene**	**Degree**	**Gene**	**Degree**	**Gene**	**Degree**	**Gene**	**Degree**
Fos	43	Calb2	27	Cd44	23	Hmgcr	18
Timp1	32	Apoe	26	Itgam	21	Syt1	18
Ccl2	31	Ptprc	26	Cacna1a	21	Cd86	18
Vim	31	Ifng	25	Gad1	20	Srebf1	17
Igf1	28	Tgfb1	23	Sst	20	Hmox1	17

### Prediction of transcription factors targeting the hub DEGs

The top 5 hub DEGs (FOS, TIMP1, CCL2, VIM and IGF1) were chosen for the prediction of TFs (Table 1). Based on the information of the Cytoscape software, 27 predicted TFs and 136 predicted motif were identified. The top 5 TFs (Tfam, Polr2a, Hoxc11, Mybl1 and Fosl2) and the top 3 motifs of each TF were all list in Table 2.

**Table 2:** Transcription factors targeting the top 5 hub DEGs.

**Transcription factors**	**Enriched motif ID**	**NES**	**Motif**	**Name of the predicted target**
Tfam	hdpi-HIST2H2BE	5.538	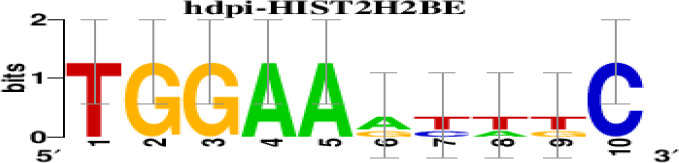	CCL2 IGF1
	hdpi-TFAM	4.329	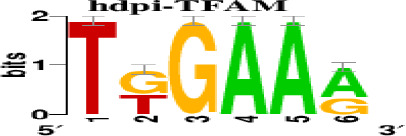	
	hdpi-LAS1L	4.329	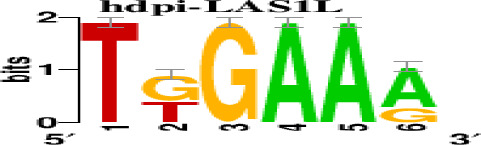	
Polr2a	factorbook-TBP	5.489	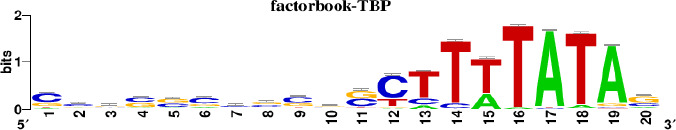	VIM FOS IGF1
	stark-STATAWAWRSVVV	3.920	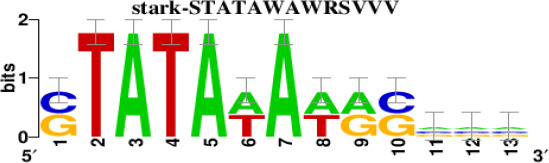	
	swissregulon-TBP.p2	3.456	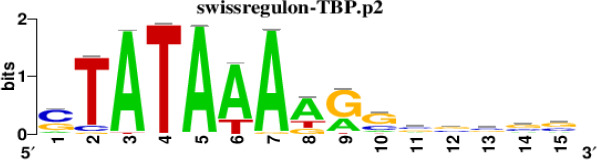	
Hoxc11	taipale-NGTCGTWAAAN-Hoxc11-DBD	5.444	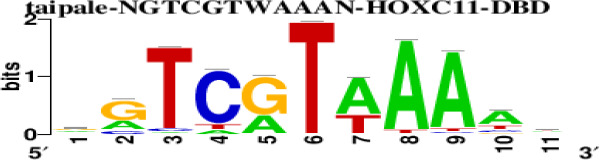	VIM FOS
	taipale-GTCGTAAAN-Hoxd9-DBD	5.153	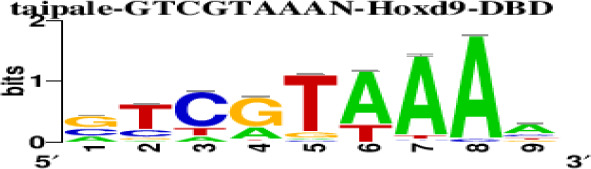	
	tiffin-TIFDMEM0000083	4.946	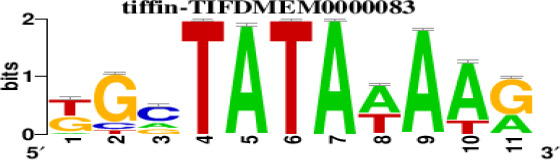	
Mybl1	flyfactorsurvey-ovo_SANGER_5_FBgn0003028	5.296	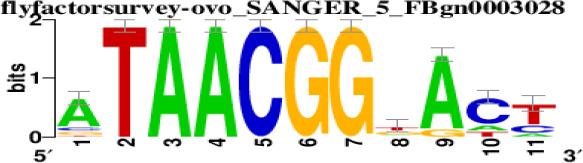	VIM FOS IGF1
	taipale-NNAACCGTTNN-MYBL1-DBD	5.212	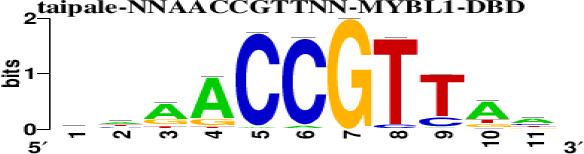	
	flyfactorsurvey-ovo_SOLEXA_5_FBgn0003028	5.040	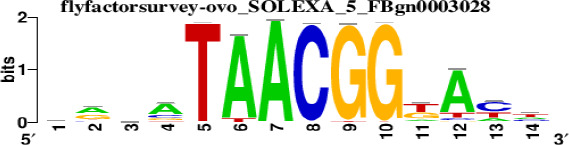	
Fosl2	yetfasco-1781	4.882	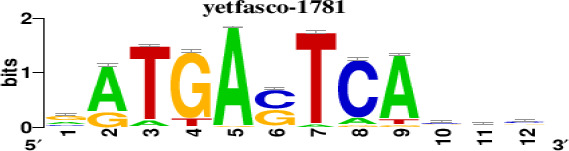	VIM IGF1 TIMP1
	factorbook-AP1	4.684	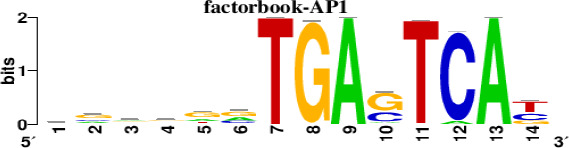	
	yetfasco-2094	4.379	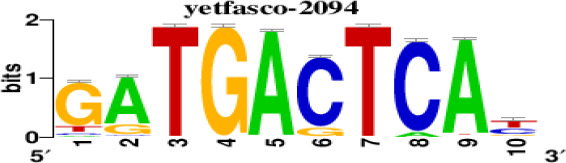	

NES; Normalized enrichment score of the motif.

### Overexpression of transcription factor A, mitochondrial (TFAM) promoted functional recovery after SCI by targeting IGF1

**Figure 5:**
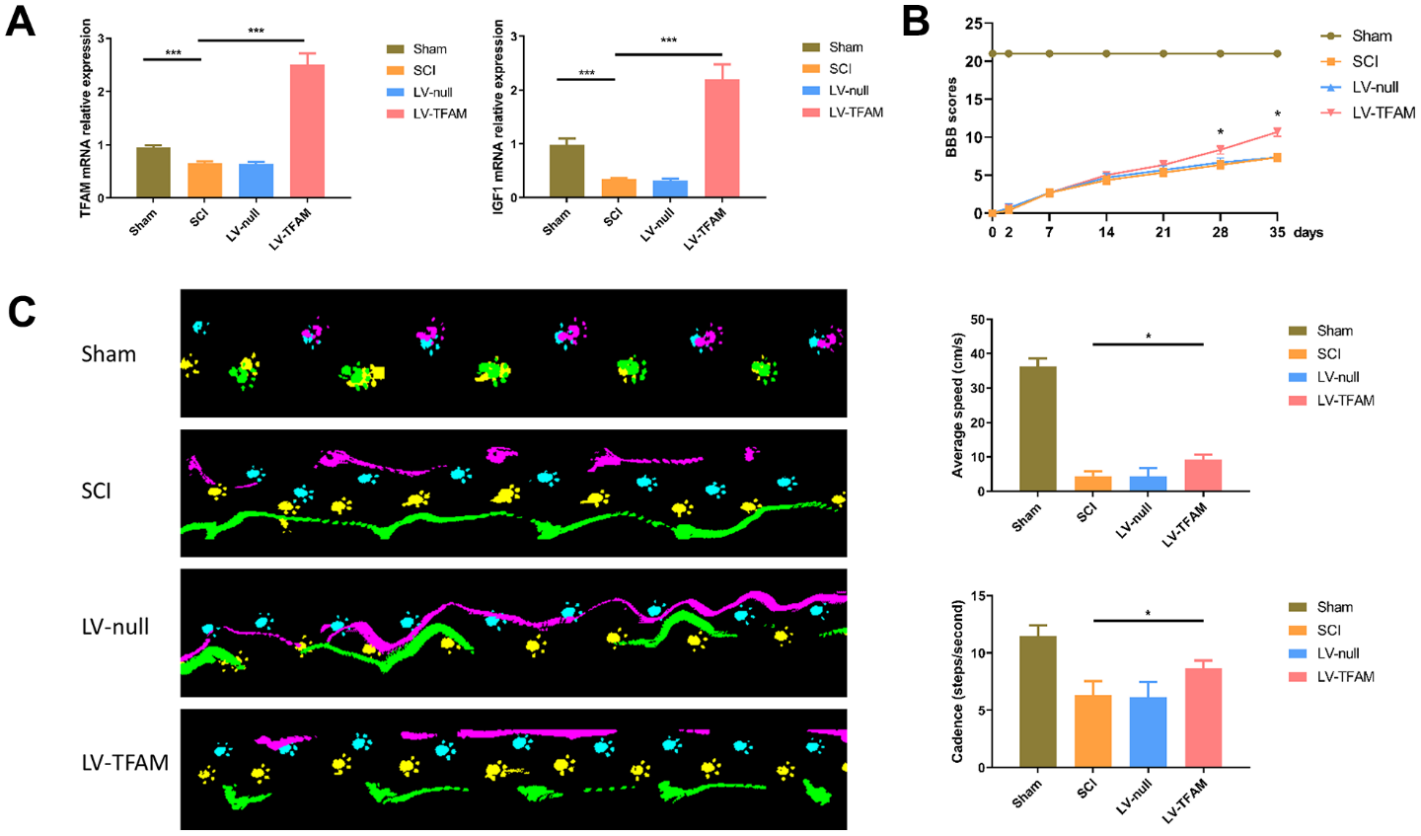
**TFAM might regulate gene transcription of IGF1 and effected alterations in the function recovery of rats after SCI.** (**A**) IGF1 and TFAM was significantly down-regulated after SCI. (**B**) BBB scale was performed for assessing motor recovery of rats hind limbs after SCI. (**C**) Gait footprints were recorded at 35 days post-injection and there were significant improvement in the TFAM-overexpression group in average speed and cadence (steps/second) compared with SCI group.

We chose IGF1 and its predicted transcription factor, TFAM for the further study. To confirm the expression of IGF1 and TFAM, we performed qRT-PCR, and the results detected that IGF1 and TFAM was significantly down-regulated after SCI (Figure 5A). We then locally injected LV-TFAM into the injured spinal cord, and the qRT-PCR results showed that IGF1 and TFAM were significantly up-regulated in LV-TFAM group. At the time points of 2, 7, 14, 21, 28 and 35 days post-injection, Basso, Beattie and Bresnahan (BBB) scale was performed for assessing motor recovery of rats hind limbs after SCI. and the results showed that after overexpression of TFAM, the BBB scores were clearly increased (*P* < 0.05) (Figure 5B). At 35 days post-injection, motor recovery after SCI was assessed using the Noldus CatWalk XT system. These observations demonstrated that there were significant differences between the injury group and TFAM-overexpression group in average speed and cadence (steps/second). The rats in the LV- TFAM group displayed faster speed and cadence, possibly indicative of motor recovery (Figure 5C). In all, the results showed a significant recovery in the TFAM-overexpression group compared to the injury group.

## DISCUSSION

SCI is known as a devastating neurological disorder that can lead to immediate sensorimotor and autonomic dysfunction below the lesion level [19, 20]. More and more researchers are focusing on this field, however, they have not yet succeeded in creating an adequate therapy concept that achieves at least partial remission and thereby restoring their function [21]. After the immediate and acute injury of spinal cord, including the trauma itself, the laceration or contusion of local cells, vessels and connective tissue, the subsequent intermediate phase will occur the further damaging to tissue [22]. The intermediate phase of SCI has the characteristic of the sustained formation and maturation of the glial scar and axonal sprouting. The glial scar has been considered as the main cause of the failure of spinal cord regeneration. Therefore, there is an urgent need for exploring the underlying mechanism under the intermediate phase of SCI.

In the present study, we examined the mRNA expression profiles and analyzed it using a comprehensive bioinformatic analysis. We found that 416 genes were significantly differentially expressed, including 210 that were upregulated and 206 which were downregulated. Intriguingly, the results of GO enrichment analysis indicated that the enriched terms were relevant to cholesterol biosynthetic process, glucuronsyltransferase activity, protein homodimerization activity, and the DEGs were significantly enriched in neuron projection, cell surface, dendrite, neuronal cell body and axon. It has been reported that the biochemical events, including lipid metabolism, occurred following traumatic injury to the mammalian spinal cord [23]. A prior investigation was conducted to assess the available data regarding a potential decrease in HDL-C levels among individuals with SCI, and the findings indicated a possible association between lower HDL-C concentrations and patients diagnosed with SCI [24]. Therefore, examining the lipid metabolism changes and their time courses after SCI is beneficial for determining the potential role of lipid metabolism in the development of SCI. Furthermore, for CC, the DEGs were significantly enriched in neuron projection, cell surface, dendrite, neuronal cell body and axon. We conclude that the DEGs may play a crucial part in the behavioral changes of neurons in the intermediate phase of SCI.

Furthermore, we subsequently identified the top 10 hub nodes with high degrees, which included FOS, TIMP1, CCL2, VIM, IGF1, CALB2, APOE, PTPRC, IFNG and TGFB1. The rapid activation of c-fos in response to a stimulus leads to the induction of its protein product, Fos, which functions as an inducible transcription factor capable of transactivating various target genes with delayed onset [25, 26]. A previous study has demonstrated that SCI elicits a rapid upregulation of c-fos and preprodynorphin expression, suggesting the involvement of c-fos in the induction of preprodynorphin expression [25]. CCL2 is known as a chemokine involving in recruiting pro-inflammatory M1 macrophages, and promotes the pro-inflammatory response in SCI [27]. A recent study also found that the delivery of human CCL2 could potentially exert a pivotal influence in mitigating motor neuron degeneration both *in vitro* and *in vivo* following SCI [28]. VIM is astrocyte cytoskeletal intermediate filament protein, and silencing of it can promote anatomical plasticity and functional recovery after SCI [29, 30]. IGF1 is able to promote the growth of CST axons in cultured neonatal CSNs [31]. APOE, a plasma protein responsible for lipid and cholesterol transportation, has the ability to modulate central nervous system responses to injury. Inhibition of microglial activation by its mimetic resulted in enhanced neurological and histological outcomes following spinal cord injury in rats [32]. Therefore, it is reasonable to hypothesize that these DEGs might play key roles during the pathogenesis of SCI.

Furthermore, we then predicted the TFs targeting the top 5 hub DEGs (FOS, TIMP1, CCL2, VIM and IGF1), and 27 predicted TFs and 136 predicted motif were identified. The top 5 TFs were Tfam, Polr2a, Hoxc11, Mybl1 and Fosl2. TFs are protein molecules that selectively bind to specific regulatory sequences on the DNA helix, thereby exerting their influence on gene transcription through trans-activation or trans-repression domains [33]. Recent advances have shown that the elucidation of genetic and epigenetic mechanisms governing the regulation of TFs and the development of novel technologies for manipulating their expression have significantly contributed to our understanding of the underlying mechanisms involved in the intermediate phase of SCI. Therefore, understanding the regulatory principles of TFs and identifying the crucial TFs that govern responses against SCI is the prerequisites to exploring the therapeutic strategy for SCI.

A previous study showed that analyzing up- and down-regulated genes separately is more powerful than analyzing all of the DEGs together [34]. This is a limitation of our study. Furthermore, in a previous study, gene expression data set GSE2599 has been analyzed preliminarily, and most results of our study are also in keeping with their earlier findings [35]. In our study, in addition to analyzing functional enrichment and pathway enrichment, we also predicted the TFs of the top 5 DEGs. These results may help us to deepen the understanding of the mechanisms of SCI.

We then chose IGF1 and its predicted transcription factor, TFAM for the further study. Previous study has suggested that overexpression IGF-1 effected beneficial alterations in the motor function recovery and alleviation of spasms after SCI in rats [36]. Furthermore, glial scar formation can be induced by releasing IGF-1 from microglia, which can trigger astrocytic activation and proliferation [37]. However, the upstream mechanism is unclear. In our study, we confirmed that IGF1 and its predicted transcription factor- TFAM were down-regulated after SCI. TFs are considered as the main regulators of gene transcription and they can interact with DNA sequences to control transcription [38]. TFAM is a multifunctional DNA-binding protein, which play a crucial part in transcriptional activation and mitochondrial DNA (mtDNA) organization [39, 40]. TFAM has the ability to engage in interactions with a transcription coactivator within a family, thereby exerting control over mitochondrial biogenesis, replication, and transcription of mitochondrial DNA. This interaction occurs through its association with peroxisome proliferator-activated receptor-γ coactivator (PGC-1α) subsequently [41, 42]. Furthermore, TFAM, combined the exercise, can leads to a prevention of skeletal muscle atrophy by regulating mitochondrial function [43]. In previous studies, TFAM has been considered to have a neuroprotective effect on SCI [44]. Our current research shows that TFAM and IGF1 are all down-regulated after SCI, and TFAM is predicted to be the targeted transcription factor of IGF1. Furthermore, overexpression of TFAM can promote the function recovery of rats after SCI. Therefore, we speculate that TFAM may regulate the gene transcription of IGF1 and effected alterations in the functional recovery of rats after SCI. In this study, we only detected the expression levels of the mRNA, and did not provide more information of the relationship between TFAM and IGF1. In subsequent experiments, more mechanism between TFAM and IGF1 need to be investigated further.

## CONCLUSIONS

In recent years, there has been growing interest in understanding the pathogenesis and underlying mechanism of the intermediate phase of SCI. In the present study, 416 DEGs were identified that were expressed in the injured spinal cord 35 days after SCI, compared to the uninjured spinal cord. Relevant genes, signaling pathways, and biological functions were identified through the execution of GO and KEGG enrichment analysis as well as PPI analysis. Furthermore, we also predicted the TFs of the top 5 key DEGs. TFAM and IGF1 are all down-regulated after SCI, and overexpression of TFAM can promote functional recovery of rats after SCI. Therefore, we speculate that TFAM may regulate the gene transcription of IGF1 and cause changes in functional recovery of rats after SCI. These results may provide a molecular target for SCI therapy and optimize therapies.

## MATERIALS AND METHODS

### Gene expression microarray data

Gene expression profile (GSE2599) was downloaded from GEO. GSE2599 is based on the Agilent GPL10787 platform (Affymetrix Rat Genome U34 Array). This data is from the spinal contusion injury site at 35 days. The dataset comprised of a total of six samples, consisting of three samples with contusion injuries and three samples without any injuries.

### Identification of DEGs

The analysis utilized TXT files as the source of raw data. The injured spinal cord and uninjured spinal cord were divided into two groups. We conducted the analysis utilizing the GEOquery and limma R packages, which are part of the Bioconductor project. Limma offers a concise overview of the linear model outcomes, conducts hypothesis tests, and appropriately adjusts *P*-values to account for multiple testing. *P*-value < 0.05 and logFC (fold change) < -1 or logFC > 1 were set as the cut-off criterion. The DEGs were identified by comparing the samples from contusion injury and uninjured individuals, and the common genes among them were determined.

### Functional and pathway enrichment analysis of the DEGs

After the identification of DEGs, we utilized the online software Database for Annotation, Visualization and Integrated Discovery (DAVID) v6.8 to detect enriched GO terms and pathway categories among the DEGs. DAVID can provide comprehensive annotations for functional and pathway interpretations. In this study, we uploaded the DEGs onto DAVID for performing the related GO and KEGG pathway enrichment analysis.

### Construction of the protein-protein interaction (PPI) network of DEGs

Search Tool for the Retrieval of Interacting Genes (STRING) database was used to further illustrate the interactive relationships among the DEGs. We utilized Cytoscape software to analyze the PPI network, focusing on interactions that have been experimentally validated and possess a confidence score exceeding 0.4. We used MCODE to screen the modules of the PPI network [45]. A network that is scale-free exhibits a distribution of vertex connectivity that adheres to a power law, characterized by a scarcity of highly connected vertices (high degree) and an abundance of vertices with low degrees The PPI network analyzed in this study exhibited the property of scale-free networks, which is indicative of a node degree distribution that closely follows a power-law distribution [46]. Three highest-degree modules were extracted, and the enrichment of functions of each module were analyzed with ClueGO.

### Prediction of transcription factor targeting the hub DEGs

TFs were characterized as proteins that bind to DNA and are essential for regulating the expression of specific genes [47]. The TFs targeting the top 5 hub DEGs were predicted with the help of iRegulon in Cytoscape software [48]. The top 5 hub DEGs were selected according to the corresponding degree using Cytoscape. The top 5 TFs and the top 3 motifs of each TF were identified for further study. The selection of the top 5 TFs was based on a TF-targets database consisting of highly reliable target genes identified through an extensive analysis of numerous cancer gene signatures using the iRegulon APP within Cytoscape. The top 3 motifs were selected according to the normalized enrichment score of the motif.

### Lentiviral production

Lentiviral vector-mediated transcription factor A, mitochondrial (LV-TFAM) and negative control (LV-null) was constructed from Genepharma (Suzhou, China). The final concentration of the lentivirus was 1 × 10^9^ TU/mL. Lentivirus were injected superficially through Hamilton microinjector at five locations around the injury site with a depth of 1 mm. Hamilton microinjector were left in place for 1 min after the injection and were then slowly withdrawn.

### Spinal cord contusion injury and lentiviral injection

The spinal cord contusion injury models were performed on female Wistar rats weighing 200–250g. These rats were divided into four groups in a random manner: a control group without any surgical intervention prior to extraction, an injured group that underwent T10 contusive spinal cord injury, and two additional groups that received lentiviral injection (LV group and LV-null) after experiencing SCI The spinal cord contusion injury model was made as described according our previous study [49]. The rats received 10μL of lentivirus (LV- TFAM) or negative control, which was slowly injected into the spinal cord at five locations (2μL was injected at each point) around the injury site using a Hamilton syringe. These animal studies have been approved by the ethical committee.

### Quantitative real-time PCR (qRT-PCR)

Total RNA was isolated with TRIzol reagent (Beijing Solarbio Science and Technology Co) at 35^th^ day after SCI. RNA quantity and quality was determined by Nanodrop 2000 (Thermo Scientific). Reverse transcription was performed using the first-strand cDNA synthesis kit (Thermo Scientific, USA) to transcribe total RNA into cDNA according to the manufacturer’s instructions. qRT-PCR was conducted using All-in-One™ qRT-PCR Detection Kit (GeneCopoeia). The qRT-PCR was performed using specific primers: TFAM (forward 5’- CCAGGAGGCTAAGGATGAGTC-3’) and (reverse 5’-CACACTGCGACGGATGAGA-3’), IGF1 (forward 5’- CAAAATGAGCGCACCTCCAA-3’) and (reverse 5’-CTTCAGCGGAGCACAGTACA-3’), GAPDH (forward 5’- GTGTTCCTACCCCCAATGTG-3’) and (reverse 5’-ATAGGGCCTCTCTTGCTCAG-3’). The qRT-PCR protocol involved an initial step of incubation at 95° C for 10 minutes, followed by 40 cycles of denaturation at 95° C for 10 seconds, annealing at 60° C for 20 seconds, and extension at 72° C for 15 seconds. Each sample was analyzed in duplicate. After that, the expression levels of genes were analyzed with the 2^-ΔΔCt^ method and normalized using U6 as the internal control.

### Quantification of the locomotor function

Locomotor assessments were performed using the Basso, Beattie and Bresnahan (BBB) locomotor test and Noldus CatWalk XT System. The hindlimb locomotor function of all rats was evaluated in an open-field walking before and 2, 7, 14, 21, 28 and 35 days after SCI according to the BBB locomotor test. Rats are allowed to move freely and are scored during 4 minutes by two observers for their ability to use their hindlimbs. The hindlimb function during locomotion (joint movements, paw placement, weight support, and fore/hindlimb coordination) were quantified according to the scale ranging from 0 to 21. The paws prints could be recorded by CatWalk XT software, and the gait analysis was performed 35 days after SCI. The rats were accessed by two observers who were blinded to this study.

### Statistical analysis

The collected data were analyzed using GraphPad Prism software (version 6.01). The mean ± standard deviation was used to express the data. Student’s t-test and two-way analysis of variance were employed to compare the differential expression levels of DEGs. A significance level of *P* < 0.05 was considered statistically significant. Bonferroni correction was applied to adjust all p-values for multiple comparisons.

### Data availability statement

Datasets analyzed during the current study are available from the corresponding author on reasonable request.
